# Seroprevalence of bovine theileriosis in northern China

**DOI:** 10.1186/s13071-016-1882-x

**Published:** 2016-11-18

**Authors:** Yaqiong Li, Zhijie Liu, Junlong Liu, Jifei Yang, Qian Li, Pengfei Guo, Guiquan Guan, Guangyuan Liu, Jianxun Luo, Hong Yin, Youquan Li

**Affiliations:** 1 State Key Laboratory of Veterinary Etiological Biology, Key Laboratory of Veterinary Parasitology of Gansu Province, Lanzhou Veterinary Research Institute, Chinese Academy of Agricultural Science, Xujiaping 1, Lanzhou, Gansu 730046 People’s Republic of China; 2 Jiangsu Co-innovation Center for Prevention and Control of Important Animal Infectious Diseases and Zoonoses, Yangzhou, 225009 People’s Republic of China

**Keywords:** ELISA, Microscopy, Prevalence, Theileriosis

## Abstract

**Background:**

Bovine theileriosis is a common disease transmitted by ticks, and can cause loss of beef and dairy cattle worldwide. Here, an indirect enzyme-linked immunosorbent assay (iELISA) based on *Theileria luwenshuni* surface protein (TlSP) was developed and used to carry out a seroepidemiological survey of bovine theileriosis in northern China.

**Methods:**

We used the BugBuster Ni-NTA His•Bind Purification Kit to purify recombinant TlSP (rTlSP), which was subsequently analyzed by Western Blotting to evaluate cross-reactivity with other pathogen-positive sera. The iELISA method based on rTlSP was successfully developed. Sera from 2005 blood samples were tested with the rTlSP-iELISA method, and blood smears from these samples were observed by microscopy.

**Results:**

The specificity of iELISA was 98.9%, the sensitivity was 98.5%, and the cut-off was selected as 24.6%. Western Blot analysis of rTlSP confirmed that there were cross-reactions with *Theileria luwenshuni*, *Theileria uilenbergi*, *Theileria ovis*, *Theileria annulata*, *Theileria orientalis* and *Theileria sinensis*. The epidemiological survey showed that the highest positive rate of bovine theileriosis was 98.3%, the lowest rate was 84.1%, and the average positive rate was 95.4% by iELISA. With microscopy, the highest positive rate was 38.9%, the lowest rate was 5.1%, and the relative average positive rate was 13.7%.

**Conclusions:**

An rTlSP-iELISA was developed to detect circulating antibodies against bovine *Theileria* in northern China. This is the first report on the seroprevalence of bovine theileriosis in northern China, and it also provides seroepidemiological data on bovine theileriosis in China.

## Background

**Fig. 1:**
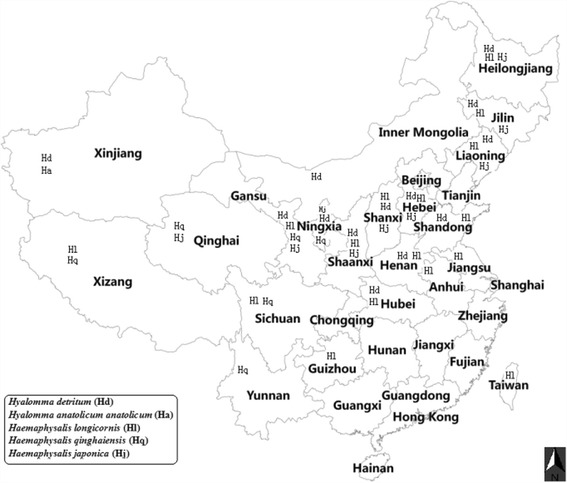
Geographical distribution of various kinds of ticks related to bovine and ovine *Theileria* spp. in China

Bovine theileriosis is a constraint to the cattle industry in many developing countries because it causes morbidity and mortality in calves and exotic cattle [1–3]. Generally, indigenous cattle have developed resistance to ticks and tick-borne pathogens in endemic areas. However, the susceptibility of exotic breeds presents a major obstacle to the improvement of cattle production and breeding [4]. The causative agents of bovine theileriosis reported in China are *Theileria annulata*, *Theileria orientalis* and *Theileria sinensis* [3, 5–7]. Five kinds of ticks, namely, *Hyalomma detritum*, *Hyalomma anatolicum anatolicum*, *Haemaphysalis longicornis*, *Haemaphysalis qinghaiensis* and *Haemaphysalis japonica*, can transmit ovine and bovine *Theileria* infection in China [8]. Of them, *Hya. detritum* and *Hya. anatolicum anatolicum* can transmit *T. annulata* [9], and *Hya. anatolicum anatolicum* can also transmit *Theileria ovis* [10]; *Haem. longicornis* and *Haem. qinghaiensis* can transmit *Theileria luwenshuni* and *Theileria uilenbergi*; *Haem. longicornis* can also transmit *T. orientalis*; and *T. sinensis* can be transmitted by *Haem. qinghaiensis* and *Haem. japonica* [6, 11, 12]. The geographical distribution of tick species linked to ovine and bovine *Theileria* spp. in China is listed in Fig. 1.

In the early phase, bovine theileriosis can be diagnosed based on observing ticks feeding on cattle, superficial lymph node enlargement, *Theileria* schizonts in lymphocytes with microscopic examination, and other clinical symptoms [3, 9]. Subsequently, *Theileria* parasites can be detected and identified in animals and ticks by polymerase chain reaction (PCR) [13, 14], reverse line blot (RLB) hybridization assay [15, 16], and loop-mediated isothermal amplification (LAMP) [17, 18]. Serodiagnosis of bovine theileriosis is performed by an indirect fluorescent antibody test (IFAT) [19–21]. Some enzyme-linked immunosorbent assays (ELISAs) to detect circulating antibodies against *T. annulata* were developed based on recombinant protein *T. annulata* surface protein (TaSP) or Tams1 [22–24]. At present, the ELISA method is widely applied as a cheap, fast and high-throughput method to screen and diagnose large numbers of clinical and field specimens.

*T. luwenshuni* surface protein (TlSP) is a transmembrane protein that contains eight antigen peptides [21–23]. It shows that TlSP owns some candidate epitopes. TlSP shares a high similarity with TaSP [25–27]. TaSP is identified as an immunodominant antigen, and it successfully used to develop and to validate a recombinant-protein-based ELISA for detecting the circulating antibodies of *T. annulata*-infected animals [22, 23, 28]. So, we concluded that we could use recombinant TlSP (rTlSP) as a diagnostic antigen in the ELISA. At present, serological epidemiological data about bovine theileriosis are deficient because of a lack of commercial or mature ELISA kits in China. Therefore, the objectives of this study were to develop an iELISA based on rTlSP, and to perform a prevalence study of bovine theileriosis with the iELISA and microscopic examination in northern China.

## Methods

### Preparation of recombinant TlSP

The functional fragment gene of TlSP was obtained from the merozoites cDNA library of *T. luwenshuni* using primers TlSP-F (5′-GGA ATT CGA TCG ACA ACG GAA TCC T-3′) and TlSP-R (5′-CCA AGC TTT AAC CCG TCA GAG TCA T-3′) [25]. The TlSP gene was inserted and expressed in the pET30a vector. The expression of rTlSP was verified by Western Blotting using anti-histidine antibody, and rTlSP was purified using the BugBuster Ni-NTA His•Bind Purification Kit (Invitrogen, Carlsbad, CA, USA).

### Western blotting

rTlSP was separated using 12% SDS-PAGE under reducing conditions and transferred to nitrocellulose membranes of 0.45 mm pore size (Amersham, Piscataway, USA). The nitrocellulose membranes were cut into 0.25 cm wide strips and blocked with 0.1 M phosphate-buffered saline (pH 7.6) containing 5% skimmed milk powder and 0.1% Tween 20 (PBST) at 37 °C for 1 h. A total of 12 different pathogen-positive sera (*T. luwenshuni*, *T. uilenbergi*, *T. ovis*, *T. annulata*, *T. orientalis*, *T. sinensis*, *Babesia bovis*, *B. major*, *B. bigemina*, *Anaplasma marginale*, *Brucella abortus*, and Bovine epizootic fever virus) were used to check the antigenicity of rTlSP. Antisera were diluted 1:100 and the secondary antibody (Anti-Bovine IgG; Sigma, St Louis, MO, USA) was diluted 1:20,000 in PBST. After three washes with PBST, positive signals were revealed by 5-bromo-4-chloro-3-indolyl phosphate/nitro blue tetrazolium (BCIP/NBT) liquid substrate system (KPL, Gaithersburg, MD, USA) for 2 min.

### Sera

Ninety-two negative sera (negative control) were collected from Chinese Holstein cattle at Lanzhou Dairy Farm, which is located in Lanzhou City, and neither ticks nor bovine theileriosis have been detected in the region for many years. Meanwhile, the whole blood samples with anticoagulant were collected and their smears were prepared. Ninety-two blood smears were negative by microscopic examination (ME); their relative sera were also negative by IFAT; and genomic DNA from 92 blood samples was negative by PCR [29]. As the 736 positive sera by IFAT (including 90 positive sera only by IFAT, and 646 positive sera by IFAT, PCR and ME), they were randomly picked from samples which were collected from local cattle in Xinjiang, an epidemic area of bovine theileriosis. Positive (*n* = 736) and negative (*n* = 92) sera were used in the next methods. The mixture of all positive sera was used as the positive control and the mixture of all negative sera was used as the negative control in the ELISA.

**Fig. 2:**
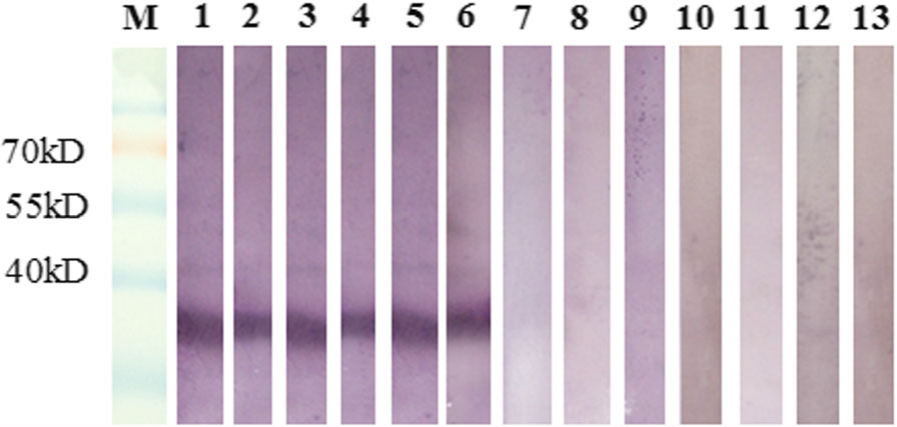
Western blotting of rTlSP. Lane M: prestained markers; Lane 1: *T. luwenshuni*; Lane 2: *T. uilenbergi*; Lane 3: *T. ovis*; Lane 4: *T. annulata*; Lane 5: *T. orientalis*; Lane 6: *T. sinensis*; Lane 7: *B. bovis*; Lane 8: *B. major*; Lane 9: *B. bigemina*; Lane 10: *A. marginale*; Lane 11: *Br. abortus*; Lane 12: Bovine epizootic fever virus; Lane 13: PBS

There are an estimated 30 million cattle in the study region [30]. The number of samples was calculated and collected according to twenty percent of the cattle population in selected sites, where the blood samples were permitted to be collected. A total of 2005 blood samples (including sera and whole blood with anticoagulant) were collected in 2011, 2012 and 2013 from 11 different provinces of northern China (Fig. 2): Xinjiang, Tibet, Qinghai, Gansu, Inner Mongolia, Shaanxi, Hebei, Henan, Shandong, Jilin, and Liaoning, most of which are epidemic areas for bovine theileriosis. The blood smears were prepared from peripheral blood and examined by microscopy. All of the serum samples were prepared and detected with iELISA, and the genomic DNA of all whole blood samples was extracted and detected by molecular methods. All sera were divided into five equal parts. These sera and their relative genomic DNA were preserved at −20 °C until utilizing them at Lanzhou Veterinary Research Institute.

### Development of ELISA and detection of samples

A previously described ELISA protocol [31] was performed with some modifications. Briefly, 96-well microplates (Nunc, Roskilde, Denmark) were coated with 100 μl/well rTlSP (5 μg/ml) in a coating buffer (0.1 M carbonate–bicarbonate buffer, pH 9.6) at 4 °C overnight. After incubation with a blocking solution (2% gelatin in PBST), the samples, blanks (PBST), standard positive and negative controls (diluted 1:100 in PBST) were distributed in duplicate. The plate was incubated with a peroxidase conjugate of monoclonal anti-bovine horseradish peroxidase IgG (Sigma, St Louis, MO, USA) (diluted 1:20,000 in PBST) at 37 °C for 1 h. After a washing step, 50 μl of O-Phenylenediamine (OPD) (Sigma Aldrich, St Louis, MO, USA) was added to each well and incubated at room temperature for 10 min. The reaction was stopped by adding 50 μl/well of 2 M H_2_SO_4_. The optical density (OD) was measured with an ELISA reader (Microplate reader Model 680, Bio-Rad, Hercules, USA) at a wavelength of 490 nm. The results are expressed as the percentage of the specific mean antibody rate (AbR%), determined using the formula: AbR% = (Sample mean OD - Negative control mean OD)/(Positive control mean OD - Negative control mean OD) × 100%. All sera samples were measured by ELISA.

### Detection of samples by microscopic examination

Thin blood smears were prepared from peripheral blood of cattle. The smears were air-dried, fixed in methanol, stained with a 10% solution of Giemsa in PBS (pH 7.2), and then subjected to microscopic analysis. One hundred fields per slide were searched for the presence of *Theileria* piroplasms.

## Results

### Cross-reactivity with hemoparasite sera

The weight of recombinant TlSP was ~38 kDa. Western blotting of rTlSP confirmed that there was specific recognition of the recombinant antigen rTlSP by the positive sera of *T. luwenshuni*, *T. uilenbergi*, *T. ovis*, *T. annulata*, *T. orientalis* and *T. sinensis*; but there was no specific recognition of rTlSP with the positive sera of *B. bovis*, *B. bigemina*, *B. major*, *A. marginale*, *Br. abortus* and Bovine epizootic fever virus (Fig. 2).

### Cut-off, sensitivity and specificity of iELISA

**Fig. 3:**
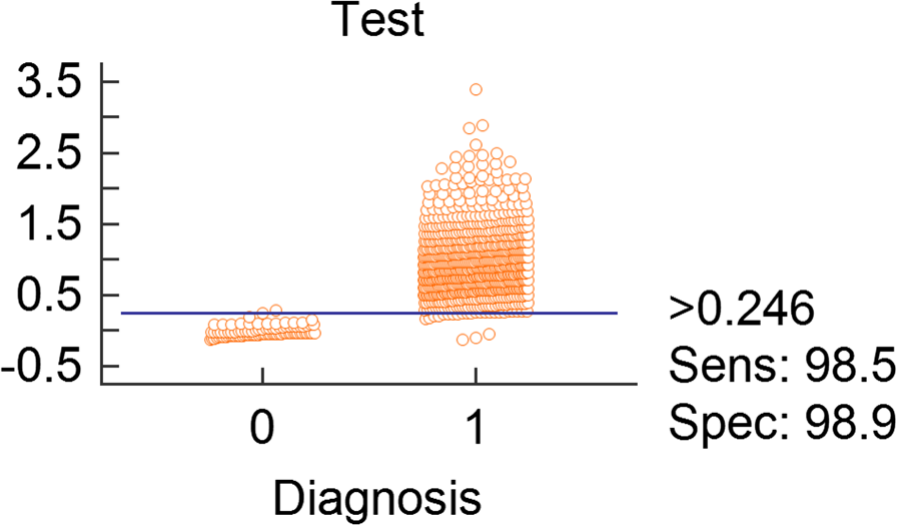
Evaluation of the positive threshold value using mean AbR of negative and positive sera (0 = negative sera; 1 = positive sera)

The cut-off, sensitivity and specificity were determined by 736 positive sera and 92 negative sera. The AbR% was calculated for each serum sample, then the receiver operating characteristic (ROC) plots (including area under the curve; AUC), cut-off and Youden’s index were evaluated with MedCalc statistical software (www.medcalc.org). Finally, the cut-off was determined to be 24.6%, corresponding to 98.9% specificity (95% confidence interval [CI]: 94.1–100%) and 98.5% sensitivity (95% CI: 97.3–99.3%) (Fig. 3); AUC 0.996, and the Youden index J 0.9742 (Table 1).

**Table 1:** Area under the ROC curve (AUC) and Youden index of negative sera and positive sera

Parameter	Value
Area under the ROC curve (AUC)	0.996
Standard Error	0.00214
95% Confidence Interval	0.989–0.999
*Z* statistic	232.120
Significance level *P* (*A*rea = 0.5)	< 0.0001
Youden index J	0.9742
Associated criterion	> 0.2460

### Serological epidemiology

The prevalence of bovine theileriosis in 11 provinces is listed in Table 2 with ELISA and microscopic examination. The epidemiology results showed that the average sera-positive rate of bovine theileriosis was 95.4% by ELISA, the highest rate was 98.3% in Xinjiang Province, and the lowest rate was 84.1% in Qinhai Province. The highest prevalence was 38.9% in Shannxi Province by microscopic examination, the lowest was 5.1% in Tibet Province, and the average was 13.7%.

**Table 2:** Prevalence of bovine theileriosis in cattle in northern China determined by iELISA and microscopic examination

Province	No. of samples	No. of positive sera by ELISA	Percentage of positive sera (%)	Percentage of positive blood smears (%)
Xinjiang	180	177	98.3	30.0
Gansu	584	527	90.2	7.0
Liaoning	111	109	98.2	27.0
Jilin	25	24	96.0	12.0
Shaanxi	54	53	98.1	38.9
Qinghai	270	227	84.1	18.1
Inner Mongolia	316	301	98.1	10.0
Hebei	30	29	96.6	13.3
Henan	60	57	95.0	30.0
Tibet	315	309	98.1	5.1
Shandong	60	58	96.6	10.0
Total	2005	1871		
Average			95.4	13.7

## Discussion

Serological surveillance of bovine theileriosis has been conducted in many countries [19, 20, 22–24, 32–35]. However, there is no previous report from China because of the lack of domestic commercial or mature ELISA kits that can detect bovine *Theileria* antibodies. In addition, the foreign ELISA kits are more expensive. Compared to other methods, the advantages of ELISA are that they are less laborious and easier to perform, and a large number of samples can be detected and screened in a short time. In addition, compared to ELISA using crude antigens (such as whole parasite antigens), recombinant-protein-based ELISAs reduce false results. Serological assays are more suitable for diagnosis in the mid and late phase of theileriosis and in carrier animals (e.g. cattle, buffalo and yaks) in which the antibody titers against *Theileria* parasites are usually higher but the piroplasm parasitemia maybe drop to undetectable levels with microscopic examination [22].

The ELISA based on rTlSP has proven to be a highly specific and sensitive assay for detecting the circulating antibodies against *Theileria* spp. in China. Three bovine *Theileria* spp. have been reported in China [3, 5, 6, 12], and their specific positive sera against each pathogen were analyzed with Western Blotting and ELISA in this study; all of them cross-reacted with rTlSP. As a comparison, there was no cross-reactivity between rTlSP and positive sera of *B. bovis*, *B. major*, *B. bigemina*, *A. marginale*, *Br. abortus* and Bovine epizootic fever virus in Western Blotting.

We chose 736 positive samples to calculate the sensitivity and specificity accurately. They included the strong positive samples (646 positive sera by IFAT, PCR and ME) and the faint positives samples (90 positive sera only by IFAT). The employ of weak positive specimen makes the cut-off value to be reasonable and can avoid false negative results.

The cut-off value resulting in both maximal sensitivity and specificity was determined by two-graph ROC analysis, ROC plots efficiency, Youden’s index and likelihood ratios [36]. The data showed that the AUC was 0.996 in ROC plots, which demonstrated the accuracy of the index. The test was reliable, and the indexes are the best performance because the area is closer to 1 [37]. Moreover, AUC is a quantitative, descriptive expression of how close the ROC curve is to the perfect one (AUC = 1.0) [38]. Youden index J was 0.9742, which indicated that, unusually, there were neither false-positive nor false-negative results from the test [39]. Compared with the positive rate with microscopy, which was <40% at the highest, iELISA based on rTlSP is suitable for detecting antibodies against bovine *Theileria* spp. Therefore, the method can estimate whether the local cattle had been infected by *Theileria* spp. before the cattle were detected. It remains to be detected by blood smears and molecular methods, such as PCR, RLB and LAMP, to diagnose whether the cattle are infected by *Theileria* spp. After the cattle infected with *Theileria* parasites recovered, most remained in a state of low parasitemia or carrier state for 3–6 months (data unpublished). Investigation of bovine theileriosis is of veterinary significance.

The epidemiological survey indicated that all the provinces investigated had a high positive rate from 84.1 to 98.3% with iELISA. In comparison, the positive rate was 5.1–38.9% by microscopic examination. Microscopic examination can diagnose *Theileria* parasites in the early and middle phase of theileriosis, whereas, in the later phase and carrier state, parasitemia tends to a low level so that *Theileria* can not be detected by microscopic examination. For example, in Tibet, the positive rate was 5.1% by microscopic examination, but up to 98.1% by ELISA.

## Conclusions

The epidemiological data showed that bovine theileriosis was widespread in northern China. Therefore, some efficient measures should be implemented for calves and exotic cattle in northern China.

## Abbreviations

BCIP/NBT: 5-bromo-4-chloro-3-indolyl phosphate/nitro blue tetrazolium
CI: Confidence interval
iELISA: Indirect enzyme-linked immunosorbent assay
IFAT: Indirect fluorescent antibody test
OD: Optical density
OPD: O-Phenylenediamine
PBST: Phosphate-buffered saline (PBS) containing 0.1 % Tween 20
ROC: Receiver operating characteristic
rTlSP: Recombinant TlSP
TlSP: *Theileria luwenshuni* surface protein
